# Crocin improves the renal autophagy in rat experimental membranous nephropathy via regulating the SIRT1/Nrf2/HO-1 signaling pathway

**DOI:** 10.1080/0886022X.2023.2253924

**Published:** 2023-09-19

**Authors:** Hongyan Liu, Hong Cheng, Hongyun Wang, Qiong Wang, Jun Yuan

**Affiliations:** a Department of Nephrology, Renmin Hospital of Wuhan University, Wuhan, China; b Renal Division, Hubei Provincial Hospital of Traditional Chinese Medicine, The Affiliated Hospital of Hubei University of Chinese Medicine, Wuhan, China; c The First Clinical College, Hubei University of Chinese Medicine, Wuhan, China

**Keywords:** Membranous nephropathy, crocin, passive Heymann nephritis, proteinuria, podocyte, Sirt1/Nrf2/HO-1 pathways

## Abstract

Membranous nephropathy (MN) is a glomerular disease. Crocin is isolated from saffron and gardenia. Its antioxidant, anti-inflammatory, anti-hyperlipidemic, anti-atherosclerotic, anti-tumor, free-radical scavenging and neuroprotective activities have been well established. We investigated the biological functions of crocin and its related mechanisms in MN. We established an experimental passive Heymann nephritis (PHN) rat model induced by anti-Fx1A antiserum. The rats were divided into sham, sham + crocin, PHN, PHN + crocin, and PHN + enalapril groups. Blood samples and kidneys of rats were collected for estimation of biochemical parameters in serum and oxidative stress indicators in kidney tissues. Histopathological changes of renal tissues were evaluated by hematoxylin and eosin, periodic acid-Schiff (PAS) and Masson staining. The podocyte number was estimated by immunohistochemistry staining of Wilms tumor type 1 (WT1). The deposition of rat anti-rabbit IgG antibodies, complement C3 and C5b-9 was detected by immunofluorescence staining. Western blotting was performed to measure the levels of Sirtuin 1 (Sirt1), nuclear factor erythroid 2-related factor 2 (Nrf2), heme oxygenase 1 (HO-1) and apoptosis-related proteins. The total cholesterol, triglycerides, creatinine, blood urea nitrogen, urine volume and urine albumin of PMN rats were significantly reduced by crocin. Additionally, crocin attenuated the renal histopathological changes. Moreover, the oxidative stress damage and podocyte loss and immune injury were relieved by crocin in PHN rats. Mechanistically, crocin administration activated the Sirt1/Nrf2/HO-1 pathways. The results provide a scientific basis that crocin could alleviate MN by inhibiting immune injury and podocyte damage through activating the Sirt1/Nrf2/HO-1 pathways.

## Introduction

Membranous nephropathy (MN) is an immune-mediated glomerular disease characterized by proteinuria, the presence of subepithelial immune complexes, the granular deposit of IgG and complement along the peripheral glomerular capillary loops and diffuse thickening of the glomerular basement membrane (GBM), and is a common cause of the nephrotic syndrome in adults [1]. In China, the incidence of MN becomes yearly more widespread, and 67.3% of elderly patients over 60 years of age are diagnosed with MN [2,3]. Notably, 33–50% of patients with nephrotic syndrome develop end-stage renal failure within 20 years of onset [4]. Approximately 75% of MN cases are idiopathic membranous nephropathy (IMN) which is mainly associated with anti-PLA2R and THSD7A [5,6], but nearly 25% are secondary to various causes, including infection, autoimmune diseases, cancers, and medications [7,8]. Currently, the common background medications for MN are glucocorticoids, cyclophosphamide, calcineurin inhibitors, and rituximab. Nevertheless, after medication, low complete remission and high recurrence rate still exits, and the side effects such as infection caused by immunosuppression require to be addressed [9]. Thus, better understanding the mechanism involving MN pathogenesis and exploring novel effective therapeutic options for treating MN are urgent.

Glomerular podocytes are terminally differentiated and highly specialized cells with GBM and the endothelium to maintain the renal blood-urine filtration barrier function [10]. Podocytes are damaged during the progress of MN due to the immune complex activating the complement cascade [11]. After the complement system is activated by immune complexes, C5b-9, also called membrane attack complex, assembles on the podocyte membrane resulting in podocyte injury [12]. The deposition of immune complexes also causes glomerular infiltration barrier disruption, extracellular matrix (ECM) accumulation and inflammatory cell infiltration, leading to heavy proteinuria and renal fibrosis [13]. Persistent podocyte injury leads to loss and death of podocytes, inducing progressive kidney damage and ultimately kidney failure [14]. Additionally, the limited proliferative capability of podocytes is a major contributor to the development of progressive glomerulosclerosis [15]. Glomerulosclerosis often suggests the presence of proteinuria [16]. Accumulating studies have illustrated that the potential mechanism of podocyte damage is associated with various physiological changes, such as oxidative stress and apoptosis [17,18]. Thus, inhibiting immune action and reducing podocyte lesion could evidently suppress renal damage and proteinuria of MN.

Crocin is a bright red, water-soluble carotenoid pigment isolated from saffron and gardenia [19]. Chemically, crocin is a crocetin digentiobiose ester (C_20_H_24_O_4_), forms crystals with a 186 °C-melting point, and is hydrolyzed by emulsion. Ingested crocin is partly metabolized to mono- and di-glucuronide conjugates [20,21]. Various pharmacological examinations have revealed that crocin possesses anti-cancer, antioxidant, anti-inflammatory, anti-hyperlipidemic, and anti-atherosclerotic activities [22–25]. Previous studies have suggested that crocin could suppress kidney damage *via* by suppressing oxidative stress and apoptosis [26–29]. However, the biological functions of crocin in MN development remain uncertain.

Heme oxygenase (HO) is the rate-limiting enzyme that degrades heme into carbon monoxide, ferritin and biliverdin. As an isoform of HO, heme oxygenase 1 (HO-1) serves as the microsomal rate limiting enzyme of heme catabolism and modulator of biological processes [30]. Accumulating evidence has indicated that HO-1 is involved in the regulation of podocyte apoptosis [31–33]. HO-1 is a downstream factor regulated by nuclear factor erythroid 2-related factor 2 (Nrf2), and activation of the Nrf2/HO-1 pathway relieves kidney injury after experimental MN by inhibiting podocyte loss [34]. Sirtuin 1 (Sirt1) is an NAD^+^-dependent deacetylase that can regulate glucose metabolism, energy homeostasis and cellular stress responses through deacetylation of diverse factors. It is found to attenuate oxidative stress, inflammation and apoptosis [35–37]. The renoprotective effect of Sirt1 has been demonstrated in diverse renal diseases [38,39]. Crocin is suggested to protect renal epithelial cells against high glucose-induced injury through activating the Sirt1/Nrf2/HO-1 pathways [40]. Rat passive Heymann nephritis (PHN) is the most preferred rodent model of human MN, and it is induced by passive administration of anti-Fx1A serum, which can bind to antigens on the podocyte [41]. Therefore, this study was designed to investigate the biological functions of crocin in MN development and detect whether the effect of crocin was mediated by the Sirt1/Nrf2/HO-1 pathways in PHN rats. Several studies have shown that angiotensin-converting enzyme (ACE) inhibitors can reduce proteinuria in patients with MN [42,43]. Enalapril is a potent, orally-active, long-acting, nonsulphydryl ACE inhibitor, can treat the proteinuria of membranous glomerulonephritis without detriment to systemic or renal hemodynamics [44]. Hence, enalapril was used as a positive control in this study. We hypothesized that crocin might protect against MN. We believe that this study would provide novel insights into clinical application of crocin in the management of MN.

## Methods

### Animals

Sprague-Dawley (SD) rats (male, 180–220 g) were obtained from Charles River Laboratories (Beijing, China) and housed under standard specific pathogen-free conditions (20 ± 2 °C controlled temperature, 50 ± 10% humidity, and a 12 h light/dark cycle) with free access to standard laboratory water and food. The study was pre-approved by the Animal Ethics Committee of Wuhan Myhalic Biotechnology Co., Ltd (No. 202206047). All experiments involving animals were implemented under the guidelines for the Care and Use of Laboratory Animals published by the National Institutes of Health.

### Establishment of the PHN model

**Figure 1.**
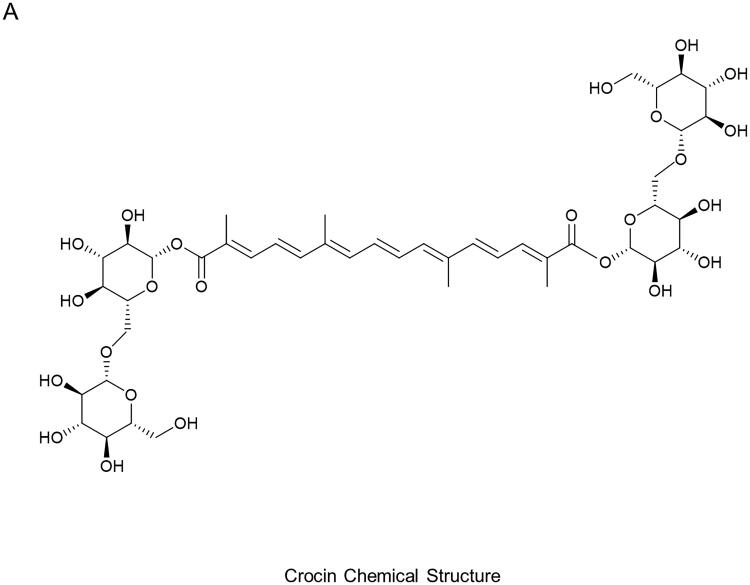
Chemical structure of crocin.

The PHN rat model was used to mimic human MN as previously documented [45]. Rats were acclimated for 3 days and then randomly divided into five groups: (a) sham, rats received a tail vein injection of normal saline (0.5 mL/100 g) once, and after 7 days, rats were given 12.6 mL/kg of distilled water by oral gavage daily for 30 days; (b) sham + crocin, rats were given a tail vein injection of normal saline (0.5 mL/100 g) once, and after 7 days, rats were injected intraperitoneally with 100 mg/kg of crocin (purity 99.41%; MedChemExpress, Shanghai, China) daily for 30 days; (c) PHN, rats were injected with a single dose of anti-Fx1A serum (0.5 mL/100 g; Probetex, Beijing, China) through tail vein, and after 7 days, rats were given 12.6 mL/kg of distilled water by oral gavage daily for 30 days; (d) PHN + crocin, rats were administrated with 0.5 mL/100 g anti-Fx1A serum *via* a single tail vein injection, and after 7 days, rats were injected intraperitoneally with 100 mg/kg of crocin daily for 30 days; (e) PHN + enalapril (40 mg/kg; MedCehmExpress): rats were administrated with 0.5 mL/100 g anti-Fx1A serum *via* a single tail vein injection, and after 7 days, rats were given 40 mg/kg enalapril daily *via* intraperitoneal injection for 30 days as positive control. Each group had 10 rats. The dose of crocin (chemical structure, Figure 1) was selected as previously reported [46]. Notably, the 24 h urine proteinuria was detected at 7 days after anti-Fx1A serum administration, and only the rats with 24 h proteinuria ≥ 100 mg were used in the study.

### Biochemical analysis

After last administration of crocin or distilled water, rats were placed in separate metabolic cages for 24 h to collect urine and the volume of urine samples was recorded. Then, urine samples were centrifuged at 1000 rpm for 10 min at room temperature, and the supernatant was stored at −80 °C. Assessment of urine albumin (ALB) was performed using a commercial kit (Jing Kang Biotech, Shanghai, China. On the next day, rats were fasted for 12 h, and then received intraperitoneal injection of pentobarbital sodium (150 mg/kg; Sigma-Aldrich, Shanghai, China). After anesthesia, blood samples were collected from the abdominal aorta and centrifuged at 3000 rpm for 15 min. The serum was collected for the analysis of ALB (Boyao Biotechnology, Shanghai, China), total protein (TP; X-Y Biotechnology, Shanghai, China), total cholesterol (TC), triglycerides (TG), creatinine (SCr), and blood urea nitrogen (BUN) (Nanjing Jiancheng Bioengineering Institute, Nanjing, China) using the commercially available kits (Table 1). Subsequently, kidneys were rapidly removed. The left kidney was excised for 100 mg, washed in ice-cold saline and homogenized in ice-cold phosphate buffer saline (PBS) with a tissue homogenizer (Sigma-Aldrich). The supernatants were centrifuged at 12,000 rpm for 30 min and then used to measure the levels of superoxide dismutase (SOD), glutathione (GSH), catalase (CAT), and malondialdehyde (MDA) with commercially available enzyme linked immunosorbent assay (ELISA) kits (Jining Shiye, Shanghai, China) (Table 2). The optical density values were measured at 450 nm using a multimode reader (Agilent, Shanghai, China). The right kidney was fixed in 4% paraformaldehyde for histopathological analysis.

**Table 1.** Baseline measurement of biochemical parameters of rats.

Biologic samples	Parameter	Sham	Sham + crocin	PHN	PHN + crocin	PHN + enalapril
Serum	ALB (g.L^−1^)	41.21 ± 1.63	41.35 ± 1.54	41.83 ± 1.09	41.56 ± 1.12	41.42 ± 0.97
	TP (g.L^−1^)	56.54 ± 1.21	56.72 ± 1.31	56.64 ± 1.27	56.59 ± 1.28	56.29 ± 1.45
	TC (mmol.L^−1^)	1.99 ± 0.21	1.97 ± 0.22	1.95 ± 0.72	1.96 ± 0.69	1.92 ± 0.76
	TG (mmol.L^−1^)	0.25 ± 0.06	0.23 ± 0.07	0.26 ± 0.07	0.24 ± 0.06	0.25 ± 0.07
	SCr (μmol/L)	38.15 ± 3.24	38.17 ± 3.26	38.16 ± 4.14	38.21 ± 4.58	38.49 ± 2.16
	BUN (mmol.L^−1^)	7.98 ± 1.32	7.99 ± 1.26	8.01 ± 1.47	7.97 ± 1.48	7.88 ± 1.91
Urine	Urine volume (mL)	20.15 ± 2.31	20.04 ± 2.35	20.18 ± 4.53	20.22 ± 4.43	20.47 ± 1.09
	Urine albumin (mg/24 h)	9.76 ± 1.64	9.81 ± 1.69	9.79 ± 3.45	9.84 ± 3.34	9.86 ± 3.41

**Table 2.** Effect of crocin treatment on biochemical parameters of rats (after drug administration).

Biologic samples	Parameter	Sham	Sham + crocin	PHN	PHN + crocin	PHN + enalapril
Serum	ALB (g.L^−1^)	42.10 ± 1.82**	42.05 ± 1.88	35.64 ± 1.05	40.68 ± 1.24^▲^	41.12 ± 1.31^▲^
	TP (g.L^−1^)	58.63 ± 1.33**	58.65 ± 1.32	45.33 ± 1.49	56.65 ± 1.79^▲▲^	57.10 ± 0.42^▲▲^
	TC (mmol.L^−1^)	1.92 ± 0.30**	1.90 ± 0.33	5.77 ± 0.86	3.15 ± 0.63^▲^	2.945 ± 0.83^▲^
	TG (mmol.L^−1^)	0.22 ± 0.07**	0.21 ± 0.08	0.91 ± 0.17	0.33 ± 0.18^▲▲^	0.24 ± 0.32^▲▲^
	SCr (μmol/L)	37.15 ± 5.41**	37.18 ± 5.35	82.44 ± 10.05	53.45 ± 7.14^▲^	49.91 ± 6.43^▲^
	BUN (mmol.L^−1^)	8.35 ± 1.25**	8.32 ± 1.27	17.55 ± 1.70	14.35 ± 1.18^▲▲^	11.75 ± 2.41^▲▲^
Urine	Urine volume (mL)	21.33 ± 3.45**	21.32 ± 3.45	42.00 ± 1.28	29.62 ± 1.18^▲▲^	26.32 ± 2.95^▲▲^
	Urine albumin (mg/24h)	10.34 ± 1.37**	10.36 ± 1.35	68.45 ± 7.66	31.88 ± 3.72^▲▲^	28.87 ± 1.68^▲▲^

*Notes*. The Sham group vs. the PHN group **p < 0.01; the PHN + crocin group or the PHN + enalapril group vs. the PHN group ^▲^*p* < 0.05; ^▲▲^*p* < 0.01.

### Histopathological analysis

The right kidney samples were fixed in 4% paraformaldehyde for 24 h, dehydrated in graded alcohol, embedded in paraffin, and then sectioned into 4 μm slices for hematoxylin-eosin (HE), periodic acid-Schiff (PAS), and Masson (Sigma-Aldrich) staining analysis. Collagen deposition and fibrotic lesions were scored semi-quantitatively through a computer-aided point-counting morphometric analysis (MetaMorph, Universal Imaging Co., Downingtown, PA).

### Immunohistochemistry (IHC) staining

Immunohistochemistry staining of Wilms tumor type 1 (WT1) was conducted to evaluate the podocyte number. Briefly, the renal cortex was fixed in 4% paraformaldehyde overnight and then processed as follows: alcohol (70, 80, 90% each for 1 h, 100% for 2 h), xylene (20 min), and then dipping wax (40 min). Next, the brains were sectioned coronally into 4 μm slices, and blocked with 1% bovine serum, 4% normal goat serum, and 0.4% Triton X-100 (Beyotime) for 30 min at room temperature. Thereafter, tissues sections were incubated with the primary antibody against WT1 (ab267377, 1:500; Abcam, Shanghai, China) overnight at 4 °C. On the following day, the sections were incubated with an HRP-conjugated secondary antibody for 1 h at room temperature after careful washing (three PBS rinses) followed by 3,3′-Diaminobenzidine (DAB; Beyotime, Shanghai, China) coloration. The number of positive cells was calculated by ImageJ software. IHC images were taken using an optical microscope (Olympus, Tokyo, Japan).

### Western blotting

Total protein was isolated from kidney cortex tissues using Radio Immunoprecipitation Assay Lysis buffer with protease phosphatase inhibitors and protease inhibitors obtained from Beyotime, and the extract was centrifuged at 10,000 ×g for 15 min to remove cell debris. Protein concentration was quantified using a BCA protein assay kit (Beyotime). Then, protein samples (20 μg/group) were separated using 10% sodium dodecyl sulfate polyacrylamide gel electrophoresis, transferred onto a polyvinylidene fluoride membrane. After blocking with 5% skimmed milk, the membrane was incubated overnight with primary antibodies against Bax (ab182733, 1:2000; Abcam), Nrf2 (#20733, 1:1000; Cell Signaling Technology), cleaved caspase-3 (#9661, 1:1000; Cell Signaling Technology), Sirt1 (ab189494, 1:1000; Abcam), Bcl-2 (ab196495, 1:2000; Abcam), β-actin (ab8227, 1:2000; Abcam), and HO-1 (ab189491, 1:2000; Abcam) at 4 °C. Then the membranes were incubated with secondary antibodies for 2 h at room temperature. The bands were visualized using an enhanced chemiluminescent reagent (Yeasen, Shanghai, China). The immunoblot images were evaluated by ImageJ software.

### Immunofluorescence staining

For immunofluorescence staining, the kidney cortex tissues were fixed with 4% paraformaldehyde, embedded in paraffin and cut into 4 μm thick sections. After being washing three times using PBS, the sections were blocked with 5% normal goat serum in 0.5% Triton X-100 for 30 min at room temperature. Subsequently, the sections were stained with fluorescein isothiocyanate (FITC)-conjugated goat anti-rat IgG (ab6840, 1:1000; Abcam), rabbit anti-rat C3 (NBP1-32080, 1:200; Novus Biologicals, Shanghai, China) and mouse anti-rat C5b-9 (sc-66190, 1:500; Santa Cruz Biotechnology, Shanghai, China) overnight at 4 °C, followed by goat anti-mouse IgG H&L (Alexa Fluor 488). The sections were observed under a confocal fluorescence microscope (Olympus). Fluorescence intensity was measured in five randomly selected fields of six sections by ImageJ.

### Statistics analysis

Independent experiments were performed in triplicate. The data were analyzed using GraphPad Prism 8.0 software (GraphPad Inc, San Diego, CA) and expressed as the mean ± standard deviation (*SD*). The normality distribution and the homogeneity of variance were evaluated using the Shapiro-Walk test and the Levene’s test, respectively. The value of *p* > 0.05 indicated that the assumption of normality of data and homogeneity of variance was consistent, and further parameter testing could be performed. The kidney weight/body weight ratios, the percentage of PAS-positive areas, the percentage of Masson-positive areas, the WT1-positive cells/glomerulus, the expression levels of Bax, cleaved caspase-3, Bcl-2, Sirt1, Nrf2 and HO-1, and the fluorescence intensity of IgG, C3 and C5b-9 were compared using one-way analysis of variance (ANOVA) followed by the Tukey’s *post hoc* analysis. The value of *p* < 0.05 was considered statistically significant.

## Results

### Functions of crocin in renal functions and oxidative damage in PHN rats

As shown in Table 1, the baseline measurement of ALB, TP, TC, TG, SCr and BUN levels in serum along with urine volume and urine albumin before PHN modeling showed no significant difference among five groups. However, after PHN modeling, remarkable alterations were observed in ALB, TP, TC, TG, SCr and BUN levels in serum along with urine volume and urine albumin in the PHN group compared with those in the sham group. Specifically, PHN rats exhibited obviously increased TC, TG, SCr and BUN levels in serum along with urine volume and urine albumin; instead, ALB and TP levels in serum in the PHN group showed a significant decrease (*p* < 0.01). However, administration of crocin limited the PHN-induced alterations on these biochemical parameters of rats (Table 2) (*p* < 0.05 and *p* < 0.01). Then, we evaluated the effect of crocin on oxidative stress. As shown in Table 3, activities of SOD, GSH and CAT were significantly decreased and MDA content was significantly augmented in PHN rats compared to those in the sham groups (*p* < 0.01), whereas crocin administration significantly increased SOD, GSH and CAT content as well as reduced MDA content in the kidneys of PHN rats (*p* < 0.05 and *p* < 0.01). Moreover, crocin had a similar effect regarding the enalapril (positive control). Collectively, these data show that crocin ameliorates PHN-induced renal functions and prevents oxidative stress in PHN rats.

**Table 3.** Effect of crocin treatment on the oxidative stress level of kidney tissues in rats.

Homogenate	Parameter	Sham	Sham + crocin	PHN	PHN + crocin	PHN + enalapril
Kidney	SOD (U.mg^−1^)	73.85 ± 0.58**	73.83 ± 0.61	34.35 ± 0.71	51.53 ± 0.75^▲^	55.10 ± 0.49^▲^
	GSH (U.mg^−1^)	155.04 ± 2.73**	155.02 ± 2.77	64.63 ± 2.08	92.66 ± 3.24^▲▲^	100.23 ± 3.79^▲▲^
	CAT (U.mg^−1^)	3.82 ± 0.91*	3.77 ± 0.95	1.77 ± 0.84	2.65 ± 0.64^▲^	2.96 ± 0.25^▲^
	MDA (mmol.mg^−1^)	1.43 ± 0.07**	1.41 ± 0.09	9.56 ± 0.56	2.67 ± 0.54^▲▲^	2.21 ± 0.43^▲▲^

*Notes*. The Sham group vs. the PHN group **p < 0.01; the PHN + crocin group or the PHN + enalapril group vs. the PHN + saline group ^▲^*p* < 0.05; ^▲▲^*p* < 0.01.

### Functions of crocin in renal morphology and histopathology

**Figure 2.**
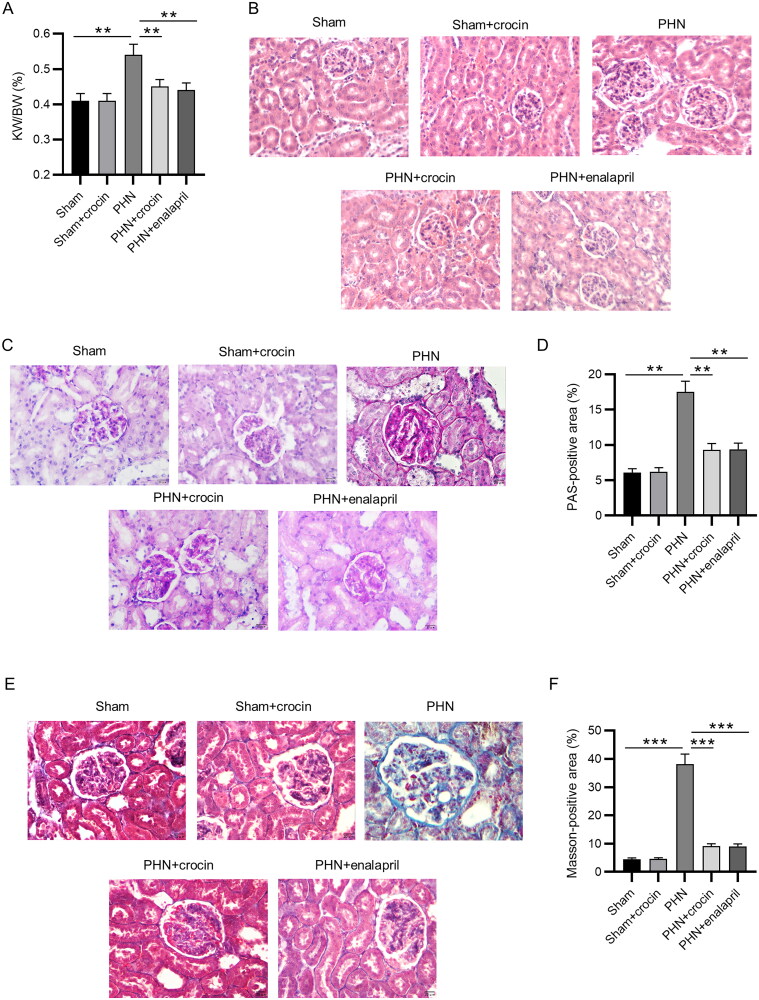
Effects of crocin on renal morphology and histopathology in PHN rats. (A) Ratio of kidney weight/body weight. (B–F) Renal tissues were investigated by staining with HE (scale bars 50 μm), PAS (scale bars 20 μm), and Masson (scale bars 50 μm). The percent of PAS-positive area and Masson-positive area was semi-quantitatively analyzed by Image-Pro Plus 6.0. *N* = 6. Data are represented as mean ± *SD* from independent groups. **p < 0.01; ***p < 0.001.

Next, we evaluated the effect of crocin on morphological and histopathological changes in PHN rats by assessing kidney weight/body weight ratio (KW/BW) and glomerular and tubular structures. As shown in Figure 2(A), PHN induction resulted in a significant increase in KW/BW ratio (*p* < 0.01), whereas crocin administration significantly decreased KW/BW ratio of PHN rats (*p* < 0.01). The results of HE staining showed uniform and consistent glomerular capillary lumen, intact basement membrane of epithelial cells and no inflammatory cell infiltration in the renal interstitium of the sham groups. However, PHN induction resulted in inflammatory cell infiltration, basement membrane thickening, glomerular hypertrophy and deformity, and tubular lumen dilatation. Notably, crocin administration decreased the expansion of glomeruli and inflammatory cell infiltration (Figure 2(B)). Then, we used PAS-stained sections to analyze GBM. The representative photomicrographs of PAS-stained kidney tissues showed that compared with the sham groups, the PHN group exhibited significantly thicker GBM (*p* < 0.01), whereas crocin administration significantly decreased the thickness of GBM in PHN rats (*p* < 0.01) (Figure 2(C,D)). Tubulointerstitial fibrosis is the primary manifestation at the end stage of MN. The blue area represented the degree of renal fibrosis in the Masson-stained kidney tissues. PHN induction led to a significant increase in the blue-positive area (renal fibrosis) (*p* < 0.001), whereas crocin administration significantly abolished the PHN-induced promotion (*p* < 0.001) (Figure 2(E,F)). The effect of crocin was similar with enalapril (positive control). These results show that crocin improves the histopathological changes of the kidneys in PHN rats.

### Functions of crocin in podocyte loss

**Figure 3.**
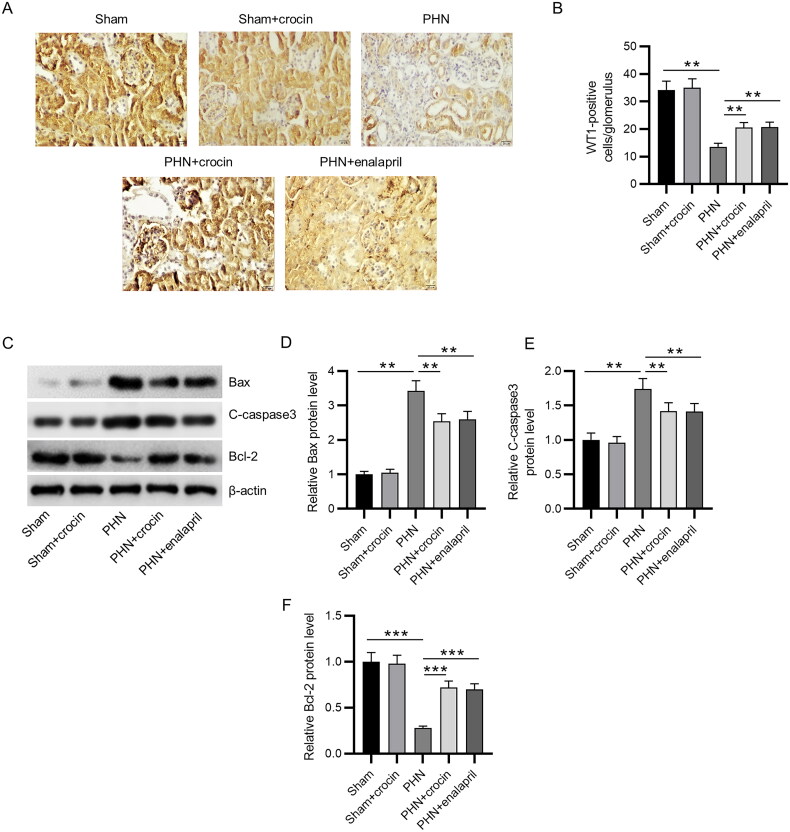
Effects of crocin on podocyte loss in PHN rats. (A–B) Immunohistochemistry staining of WT1 was performed to estimate podocyte number in the glomerular (scale bars 20 μm) (*N* = 6). (C–F) Western blotting of Bax, cleaved caspase-3 and Bcl-2 protein levels in renal tissues (*N* = 3). Data are represented as mean ± *SD* from independent groups. **p < 0.01; ***p < 0.001.

WT1 is identified as a highly expressed specific marker in mature podocytes [47]. As immunohistochemistry revealed, the number of WT1-positive cells was significantly decreased in PHN rats (*p* < 0.01), whereas administration of crocin significantly limited such loss (*p* < 0.01) (Figure 3(A,B)). Then, we measured the expression of apoptosis-related proteins, including Bax, cleaved caspase-3 and Bcl-2 in kidney tissues of rats from all groups. The results of western blotting demonstrated that the protein levels of Bax and cleaved caspase-3 were increased and the protein level of Bcl-2 was decreased after PHN induction (*p* < 0.01 and *p* < 0.001), whereas crocin administration significantly inhibited the activated apoptosis pathway by suppressing the elevation of Bax and cleaved caspase-3 protein level and the reduction of Bcl-2 protein level (*p* < 0.01 and *p* < 0.001) (Figure 3(C–F)). The anti-apoptotic activity of crocin was similar to that of enalapril. Taken together, crocin administration could effectively ameliorate podocyte injury in glomerulus of PHN rats by reducing apoptosis.

### Functions of crocin on the deposition of immune complexes in PHN rat kidney

**Figure 4.**
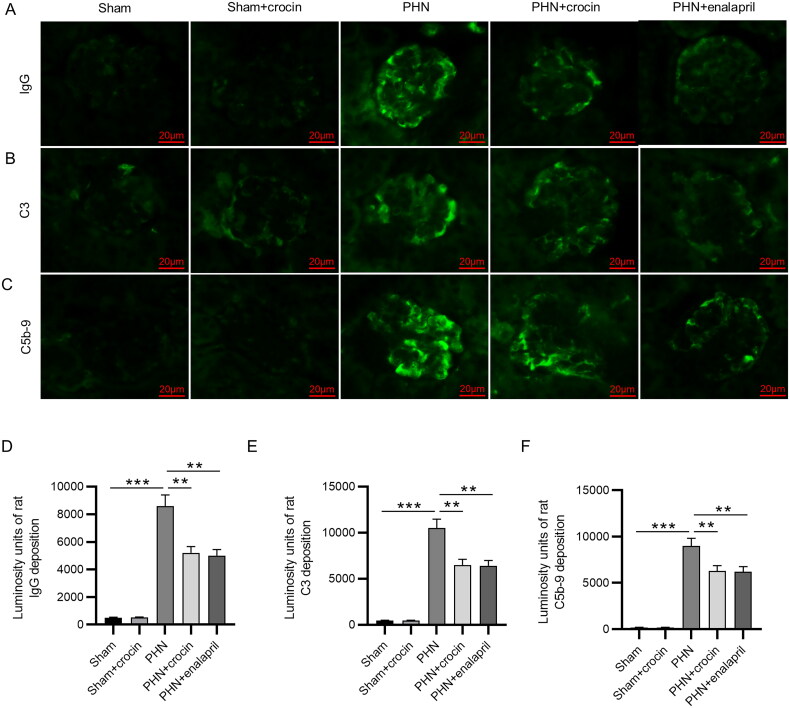
Effects of crocin on the deposition of immune complexes in PHN rat kidney. (A–C) Kidney sections in each group were detected for IgG, C3 and C5b-9 by immunofluorescence staining (scale bars 20 μm). (D) Quantification of fluorescence intensity of IgG. (E) Quantification of fluorescence intensity of C3. (F) Quantification of fluorescence intensity of C5b-9. *N* = 6. Data are represented as mean ± *SD* from independent groups. **p < 0.01; ***p < 0.001.

According to the pathogenesis of rat PHN model, the antibodies in rat anti-Fx1A antiserum occur in the kidney and recognize he rat autologous antigen megalin, which exists on podocyte and tubular epithelia, then the rat autologous antibodies recognize rabbit IgG and deposit to form an immune complex [48,49]. The PHN rats displayed pronounced autologous IgG deposition in glomeruli, dispersing along the capillary wall. The fluorescence intensity analysis revealed that PHN induction significantly increased rat IgG deposition (*p* < 0.001), which was diminished remarkably by crocin (*p* < 0.01) (Figures 4(A) and 4(D)). The complement system plays an important role in disease progression, such as deteriorating the glomerular filtration barrier and inducing renal fibrosis. In human MN, C3 and C5b-9 depositions in the kidney are typical, and they are also found in PHN animal kidneys [41,50]. As shown in Figure 4(B,C), pronounced and scattered C3 and C5b-9 deposited in both glomeruli and renal tubules in the PHN rats. By contrast, crocin treatment significantly decreased C3 and C5b-9 deposition (*p* < 0.01) (Figure 4(E,F)). Crocin had the similar protective effect against immune injury in PHN rats with enalapril.

**Figure 5.**
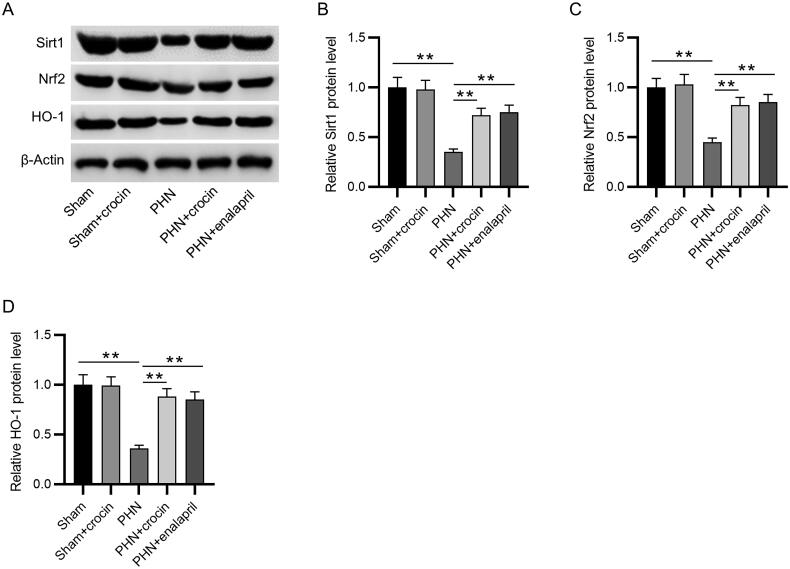
Effects of crocin on Sirt1/Nrf2/HO-1 pathways in PHN rats. (A–D) The protein expression of Sirt1, Nrf2, and HO-1 were evaluated by western blotting. *N* = 3. Data are represented as mean ± SD from independent groups. **p < 0.01.

### Functions of crocin in the Sirt1/Nrf2/HO-1 pathways in PHN rats

To investigate the potential mechanism associated with the protective effect of crocin, the protein levels of Sirt1, Nrf2 and HO-1 were measured by western blotting. The results showed that their protein levels in kidney tissues of PHN rats were significantly decreased (*p* < 0.01). However, administration of crocin could reverse their levels in PHN rats (*p* < 0.01) (Figure 5(A–D)). Crocin had the similar effect with enalapril. These results showed that crocin administration activates the Sirt1/Nrf2/HO-1 pathways in PHN rats.

## Discussion

MN can contribute to end-stage renal disease. The current medications for MN treatment develop serious side effects [51,52]. Therefore, exploring more effective targeted drugs for renal injury in MN is necessary. Crocin has been found to exert renoprotective effect in various experimental models [26–28]. This study was designed to investigate the biological functions and related mechanisms of crocin in PHN rats with enalapril as a positive control.

Clinical manifestations of MN include proteinuria, edema, hypoalbuminemia and hyperlipidemia [53]. In diabetic rats, crocin reduces serum creatinine levels, BUN, proteinuria, triglycerides and total cholesterol with concomitant increase in urinary creatinine clearance, improving kidney functions [28,54–56]. Additionally, in animal models of unilateral renal ischemia reperfusion injury, crocin pretreatment improves renal injury, as evidenced by reduced serum creatinine, BUN and proteinuria and enhanced creatinine clearance [27]. Moreover, in rats with nephropathy, crocin blocks diabetic nephropathy as indicated by decreased albumin and enhanced creatinine clearance [57]. Here, we found that administration of crocin effectively ameliorated renal injury as evidenced by decreased total cholesterol, triglycerides, creatinine, BUN as well as urine volume and albumin, and increased albumin and total protein.

Formation of antigen-antibody immune complexes in the subepithelial region of the GBM, which is manifested by GBM thickening, is a primary cause of MN pathogenesis [6]. Additionally, severe renal fibrosis occurs in the late stage of PHN [58]. Here, PAS staining and Masson staining showed that crocin reduced the thickness of GBM and renal fibrosis in PHN rats, and immunofluorescence staining revealed that crocin decreased the deposition of IgG, C3 and C5b-9. Podocyte damage may induce proteinuria [59]. WT1 plays a critical role in podocyte differentiation and is well described in podocyte health [60,61]. Here, we found that crocin abolished the PHN-induced inhibition in glomerular podocytes, which was presented as WT1-positive cells. Podocyte loss was attributed to cell apoptosis as indicated by decreased Bax and cleaved caspase-3 protein levels and increased Bcl-2 protein level. Moreover, the subsequent responses following immune complex deposits, including oxidative injure and inflammation, also appear to be central to the pathogenesis of MN [62]. This study demonstrated that crocin increased SOD, GSH and CAT levels and reduced MDA level and inflammatory cell infiltration in kidney tissues of PHN rats.

The antioxidant effect of Nrf2 is revealed in MN [63]. Crocin is shown to enhance Nrf2 and HO-1 levels in db/db mice and play anti-oxidant and anti-inflammatory roles [64]. Additionally, crocin increases Nrf2 and HO-1 expression in cellular models of diabetic nephropathy [40]. Here, we found that crocin limited the suppressive impact of PHN induction on Nrf2 and HO-1 activation. In the kidney, Sirt1 is widely expressed in tubular cells and podocytes, and its overexpression and deletion experiments in experimental models illustrate its protective role in the kidney by regulating metabolism, inflammation, and oxidative stress [65,66]. Crocin is reported to increase Sirt1 expression in diverse experimental models, including depression model, diabetic nephropathy model, and myocardial ischemia/reperfusion model [40,67,68]. Here, we found that crocin reversed the PHN-stimulated inhibition in Sirt1 expression.

In conclusion, this study demonstrates that crocin improves kidney functions of PHN rats by attenuating immune injury and podocyte damage through activating the Sirt1/Nrf2/HO-1 pathways. These results suggest that crocin is a promising therapeutic option for MN treatment.

There are limitations to this study. First, experiments were performed at a single time point; effects of crocin at additional time points are required investigation. Second, although we explored the mechanism of crocin on MN through animal experiments, the inhibitors of Sirt1/Nrf2/HO-1 pathways are required to further demonstrate the protective effects of crocin on podocytes. Third, the effect of crocin should be detected in more species before clinical practice. Finally, due to limited funding, we did not conduct transmission electron microscopy assay, which can demonstrate more the renal pathology and the impact of crocin treatment.

## Data Availability

The datasets used or analyzed during the current study are available from the corresponding author on reasonable request.
